# Quantitative surveillance of shiga toxins 1 and 2, *Escherichia coli* O178 and O157 in feces of western-Canadian slaughter cattle enumerated by droplet digital PCR with a focus on seasonality and slaughterhouse location

**DOI:** 10.1371/journal.pone.0195880

**Published:** 2018-04-12

**Authors:** Sarah-Jo Paquette, Kim Stanford, James Thomas, Tim Reuter

**Affiliations:** 1 Alberta Agriculture and Forestry, Lethbridge, Alberta, Canada; 2 University of Lethbridge, Lethbridge, Alberta, Canada; Cornell University, UNITED STATES

## Abstract

Often *Escherichia coli* are harmless and/or beneficial bacteria inhabiting the gastrointestinal tract of livestock and humans. However, Shiga toxin-producing *E*. *coli* (STEC) have been linked to human disease. Cattle are the primary reservoir for STEC and STEC “super-shedders” are considered to be a major contributor in animal to animal transmission. Among STEC, O157:H7 is the most recognized serotype, but in recent years, non-O157 STEC have been increasingly linked to human disease. In Argentina and Germany, O178 is considered an emerging pathogen. Our objective was to compare populations of *E*. *coli* O178, O157, shiga toxin 1 and 2 in western Canadian cattle feces from a sampling pool of ~80,000 beef cattle collected at two slaughterhouses. Conventional PCR was utilized to screen 1,773 samples for presence/absence of *E*. *coli* O178. A subset of samples (n = 168) was enumerated using droplet digital PCR (ddPCR) and proportions of O178, O157 and shiga toxins 1 & 2 specific-fragments were calculated as a proportion of generic *E*. *coli* (GEC) specific-fragments. Distribution of *stx*_1_ and *stx*_2_ was determined by comparing *stx*_1_, *stx*_2_ and O157 enumerations. Conventional PCR detected the presence of O178 in 873 of 1,773 samples and ddPCR found the average proportion of O178, O157, *stx*_1_ and *stx*_2_ in the samples 2.8%, 0.6%, 1.4% and 0.5%, respectively. Quantification of *stx*_1_ and *stx*_2_ revealed more virulence genes than could be exclusively attributed to O157. Our results confirmed the presence of *E*. *coli* O178 in western Canadian cattle and ddPCR revealed O178 as a greater proportion of GEC than was O157. Our results suggests: **I**) O178 may be an emerging subgroup in Canada and **II**) monitoring virulence genes may be a more relevant target for food-safety STEC surveillance compared to current serogroup screening.

## Introduction

*Escherichia coli* are commensal organisms found in the gastrointestinal tract of both humans and livestock and a subgroup has been linked to human diseases. Certain pathogenic *E*. *coli* cause extra intestinal illnesses (e.g. urinary tract infections) and various other groups such as Shiga toxin-producing *E*. *coli* (STEC) may cause gastrointestinal diseases [1]. All STEC produce at least one Shiga toxin [2] and can cause illness ranging from mild diarrhea to severe infections such as hemolytic uremic syndrome or hemorrhagic colitis [3, 4], and have been linked to both outbreaks and sporadic cases of disease [5].

Cattle are a major reservoir for STEC [6] and are often implicated as the origin of entry into the food chain [7]. Cattle that shed >10^4^ CFU/g of *E*. *coli* O157 have been coined ““super-shedders”” [8] and are deemed to be a major contributor to animal-animal transmissions [9]. A Scottish study found that 9% cattle were “super-shedders” but accounted for >96% of the *E*. *coli* O157 shed by the group [10]. Identifying “super-shedders” in a herd can be problematic as high levels of shedding can be intermittent with an animal originally negative for *E*. *coli* O157 becoming an O157 positive super-shedder [11] and identified O157 “super-shedders” no longer super-shedding after 6 days of testing [12]. Ultimately, super-shedding is not limited to O157 as STEC O26 “super-shedders” have been identified in the past [8].

*Escherichia coli* O157:H7 has been considered the most virulent serotype associated with outbreaks and sporadic STEC-related disease [2], but an increasing number of non-O157 STEC serogroups have been linked with human diseases [4]. In addition to O157, in 2011, the USDA-FSIS made testing mandatory in beef products for the top six non-O157 serogroups (O26, O45, O103, O111, O121 and O145) [13]. In Argentina, while O157:H7 is often implicated in human disease [4], it has not been as frequently isolated from cattle compared to reports in other countries and non-O157 STEC are more predominant [14]. In both Argentina and Germany, the serogroup O178 isolated from beef cattle has been linked increasingly with human disease [5]. Both *stx*_1_ and *stx*_2_ have been found in O178 isolates, although *stx*_2_ is more common, with O178 part of a group of virulent LEE-negative STEC [5, 14, 15].

Currently, conventional PCR assays are used to monitor non-O157 STEC by amplifying gene fragments to detectable concentrations. Novel technologies, like droplet digital PCR (ddPCR) have been reported to be an improvement compared to real-time PCR assays, offering absolute quantification without the need for internal standards [16].

A previous two-year study by our laboratory characterized the top-7 serogroups present in western Canadian cattle before slaughter [17]. The first objective of the current study was to examine the presence of *E*. *coli* O178 in Canadian cattle based on our sampling pool. Secondly, to develop a ddPCR assay to enumerate total *E*. *coli* specific gene fragments to compare serogroup and toxin specific gene fragments for O157, O178, *stx*_1_ and *stx*_2_ as a proportion of the total *E*. *coli* population and thirdly, examine distribution of *stx*_1_ and *stx*_2_ versus O157 to correlate high enumerations of *stx*_1_, *stx*_2_ and O157.

## Materials and methods

### Bacterial DNA

Over a two-year period, metagenomic DNA was isolated after enrichment from 1,773 pooled feces samples collected from transport trailers containing up 45 beef cattle at 2 slaughterhouses (**A** = 928 and **B** = 845) located 200 km apart in Alberta, Canada. In short, fecal samples were mixed by hand and a 15 g subsample was then mixed with 135 mL EC broth (EMD Millipore) using a Seward Model 400 stomacher (Cole-Palmer) at 230 rpm for 1 min. Fecal suspension (10 mL) was then transferred to a sterile culture tube and incubated for 6 h at 37°C. A 1 mL aliquot of the enriched culture was centrifuged at 8,000 X g for 10 min before extraction of DNA from the pellet using the NucleoSpin Tissue Kit (Macherey-Nagel) as described previously [17].

### O178 screening by conventional PCR

PCR was performed on metagenomic DNA on samples of individual enrichments using a multiplex PCR with two standalone primer sets (200nM) targeting the O178 O-antigen gene cluster (Table 1). HotStarTaq polymerase (Qiagen) was used with following cycling conditions: initial denaturation: 95°C– 5 min, 35 cycles: 95°C– 30sec, 59°C– 45sec, 72°C and final extension: 72°C– 5 min. All PCRs were carried out with 2μl template on Bio-Rad cyclers C1000 or T100 and amplicons were visualized with GelRed stain using a Molecular Imager GelDoc-XR+ (Bio-Rad).

**Table 1:** Primer sequence and amplicon size for O178 conventional PCR screening and for Evagreen droplet digital PCR assays.

Primer	Primer Sequence (5'-3')	Amplicon Size	Reference	Assay
O178–3	CCAGAGCTAAACTCAGAGGGG	112 bp	this study	O178 Screening
O178–4	GTGTGTTGAGTGTTGGCTCA			
O178–5	TCGGACGTATTTGCTGGCGCT	138 bp	this study	O178 Screening and ddPCR
O178–6	TCTGGGGGTCATAATTCAACTGGT			
O157—F2	AGGGGTTGTATGCTCGTTGT	121 bp	[18]	ddPCR
O157—R2	TGGAACACCTTCAACTTGCTCT			
GEC—*uidA*-UAL	TGGTAATTACCGACGAAAACGGC	147 bp	[19]	ddPCR
GEC—*uidA*-UAR	ACGCGTGGTTACAGTCTTGCG			
*stx*_1_—TF	CGCAGTCTGTGGCAAGAGCGAT	260 bp	this Study	ddPCR
*stx*_1_—TR	TGCCACGCTTCCCAGAATTGCAT			
*stx*_2_—F	ACTGTCTGAAACTGCTCCTGTG	307 bp	[18]	ddPCR
*stx*_2_—R	CGCTGCAGCTGTATTACTTTCC			

GEC denotes: Generic *Escherichia coli* and *stx* denotes: shiga toxin

### Droplet digital PCR

A selected subset of 168 samples from O178-positive enrichments were screened by ddPCR for absolute quantification of O178, O157, total generic *E*. *coli* (GEC) using the beta-glucuronidase gene, *stx*_1_ and *stx*_2_. The selection included 21 samples each from Summer I (June, July and August 2013), Winter I (December 2013, January and February 2014), Summer II (June, July and August 2014) and Winter II (December 2014, January and February 2015) for both slaughterhouses. The ddPCR used the EvaGreen assay as per ddPCR Applications Guide (Bio-Rad) using 2μl of template and the following PCR (QX200 EvaGreen ddPCR Supermix, Bio-Rad) conditions: Enzyme activation: 95°C—5 min, 40 cycles: 95°C—30sec, 60°C—60sec, signal stabilization: 4°C—5 min, 90°C—5 min. The primer concentration was 100nM (Table 1). The amplicons were read on the droplet reader (QX200, Bio-Rad) and analyzed using Quantasoft software (Bio-Rad).

### Droplet digital PCR analysis

Proportion (in %) was calculated based on the total number of positive amplicons for O178, O157, *stx*_1_ and *stx*_2_ compared to total GEC amplicons (S1 Fig). *Stx*_1_, *stx*_2_ and O157 amplicon distribution was determined by plotting *stx*_1_, *stx*_2_ enumerations versus O157. For *stx*-related contrast analyses, the total amount of O157 was reduced based on a recent finding that 23% of O157 isolates in the sampling pool lacked *stx* genes (Stanford—unpublished data).

### Statistical analysis

Conventional PCR results for *E*. *coli* O178 were examined for seasonal prevalence and difference between slaughterhouses, as determined by a generalized linear mixed model (Proc Glimmix, SAS 9.3) using a binomial distribution. Numerical data generated by ddPCR for GEC, O178, O157, *stx*_1_ and *stx*_2_ were examined for normal distribution and the data were log transformed prior to analyses. Seasonality, slaughterhouse, year, and interactions were determined for GEC, O178, O157, *stx*_1_ and *stx*_2_ using a mixed linear model (Proc Mixed, SAS 9.4). *P* values < 0.05 were considered significant.

## Results

### Conventional PCR screening of metagenomic DNA for O178

Screening DNA from 1,773 enrichments identified 873 (50%) samples positive for both O178-specific PCR fragments compared to previously reported 1,378 (79%; n = 1,749) *E*. *coli* O157 PCR positives within the same sampling pool [17]. Primers specific for the O178 O-antigen gene cluster showed no cross-reaction with the top 7 STEC (data not shown). O178 was identified across all seasons at both sampling sites in Alberta (Table 2) and differed in seasonal prevalence (*P* = 0.0001) with O178 presence higher in winter compared to summer. Prevalence of O178 also differed between the two sampling locations and was lower at slaughterhouse **A** than **B**, (*P* = 0.0004). Based on these results, 21 samples from each sampling location (slaughterhouse) and season (summer and winter) were selected for subsequent ddPCR analysis.

**Table 2:** Percentage of conventional PCR *Escherichia coli* O178 positives by season and slaughterhouse from 1,773 enrichments.

		Mean (% Positives)	Std. Error	Probability	Total Positives
**Season**	**Summer**	35.6^a^	0.024	**0.0001**	181
	**Fall**	33.0^a^	0.023		150
	**Winter**	71.5^b^	0.020		276
	**Spring**	50.5^c^	0.028		266
**Slaughterhouse**	**A**	43.1^a^	0.019	**0.0004**	374
	**B**	52.4^b^	0.018		499

^a, b, c^ Means with different superscripts within category differ by probabilities shown

### Droplet digital PCR

#### Total numbers of GEC, O178, O157, *stx*_1_ and *stx*_2_

**Fig 1:**
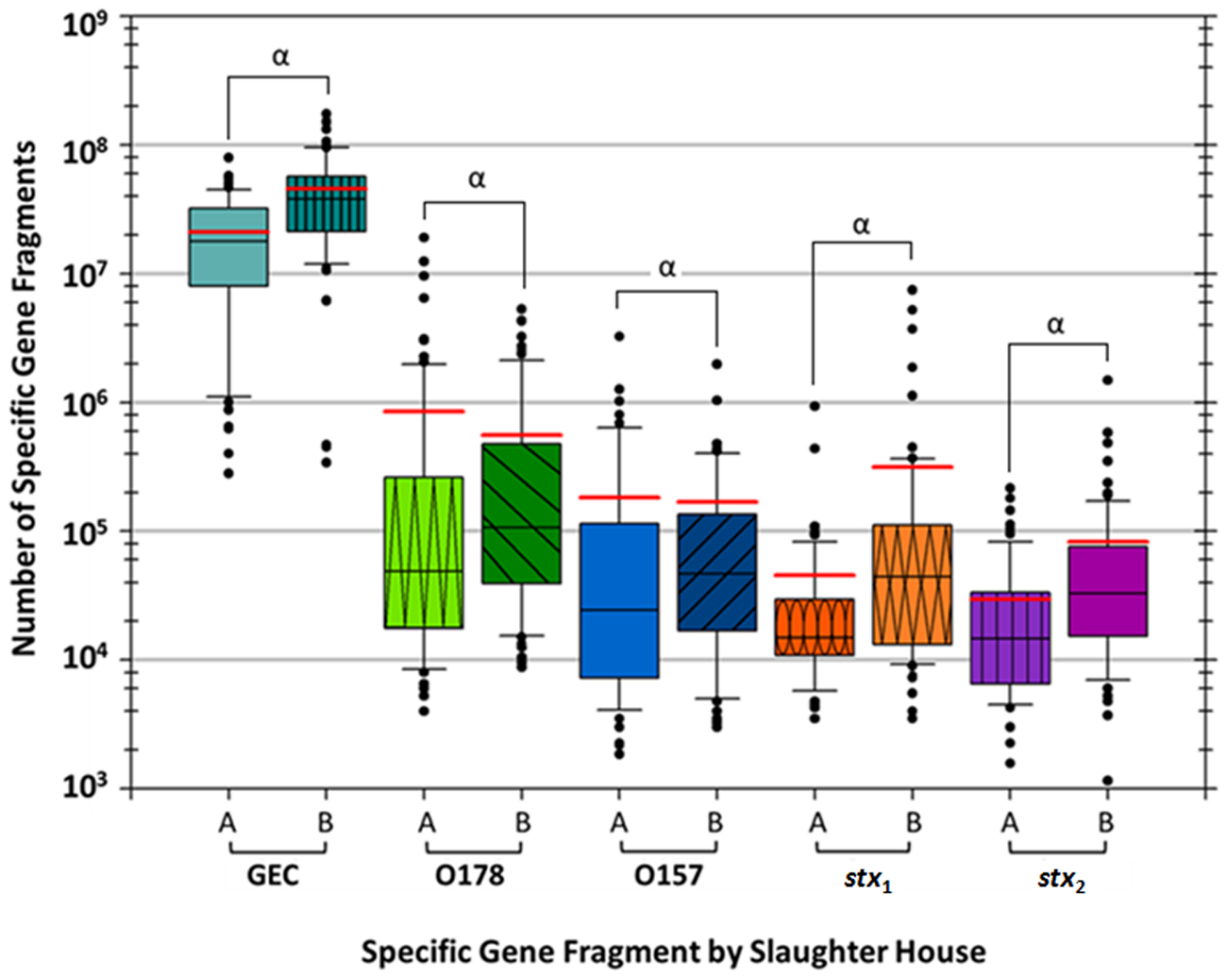
Total number of generic *E*. *coli* (GEC), O178, O157, shiga toxin (*stx*) 1 and 2 specific gene fragments in samples from slaughterhouse A or B (calculated using droplet digital PCR). In each comparison between slaughterhouse A and B there were significant differences in enumeration of gene fragments, denoted by symbol α. Note: *The red line is the average number of GEC, O178, O157, *stx*_1_ and *stx*_2_ at each slaughterhouse and the black line in the boxes is the median.

Fragments representative of GEC were enumerated in the original DNA samples and analyzed using Quantasoft (Fig 1). On average, GEC numbers were 2-times higher at slaughterhouse **B** (Fig 1) compared to **A** (*P* < 0.05). The O178 amplicons were on average 1.5-times higher at site **A** compared to **B** (*P* < 0.05), which is inconsistent with the findings of the conventional O178 PCR screening (Table 2). O157 ddPCR quantification revealed higher numbers at slaughterhouse **A** compared to **B** (*P* < 0.05) (Fig 1). Either *stx*_1_ and/or *stx*_2_ gene fragments were detected in all samples (n = 168) except four winter samples. Across all samples, *stx*_1_ and *stx*_2_ were 7- and 2.8-times higher at site **B** compared with **A**, respectively (Fig 1; *P* < 0.05).

#### Proportion of O178, O157, *stx*_1_ and *stx*_2_

**Fig 2:**
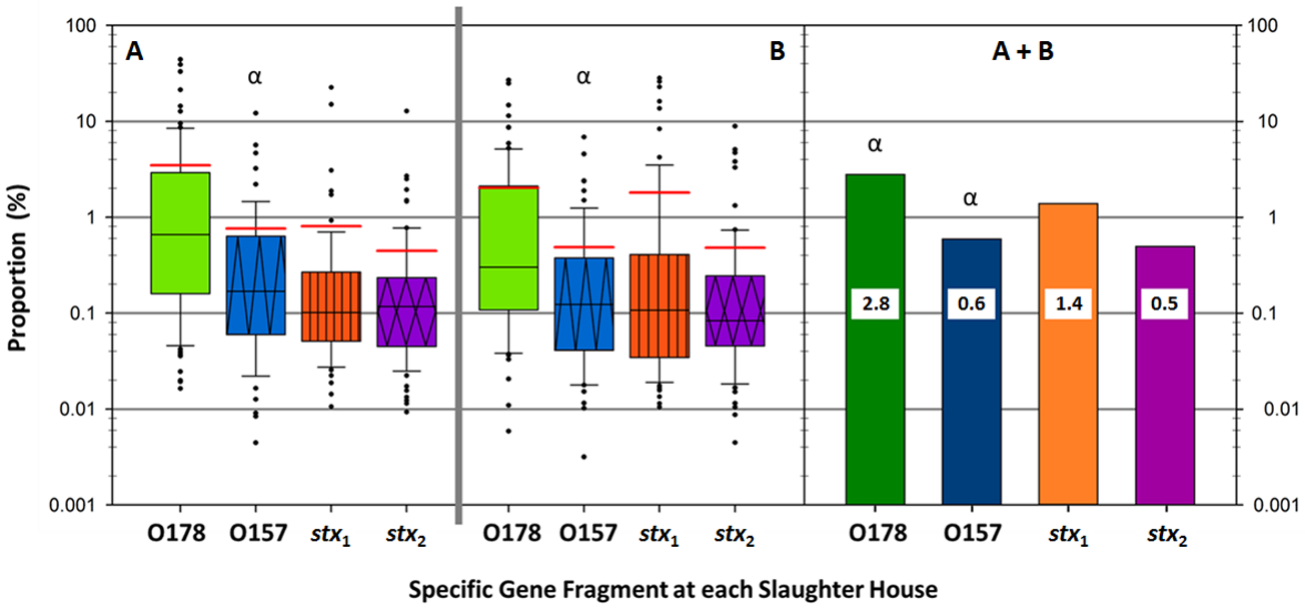
Proportion of O157, O178, shiga toxin (*stx*) 1 and shiga toxin 2 specific gene fragments in samples for A and B slaughterhouses calculated using droplet digital PCR enumerations and comparison of the average proportion of the target gene compared to generic *Escherichia coli* (GEC) in the samples. Note: *Proportions are based on the total *E*. *coli* counts. **Red line is the average proportion for the group and black line in the boxes is the median. *** Symbol: α denotes a significant difference between O157 at site A and B and between the average proportion of O178 and O157 (*P* < 0.05).

**Fig 3:**
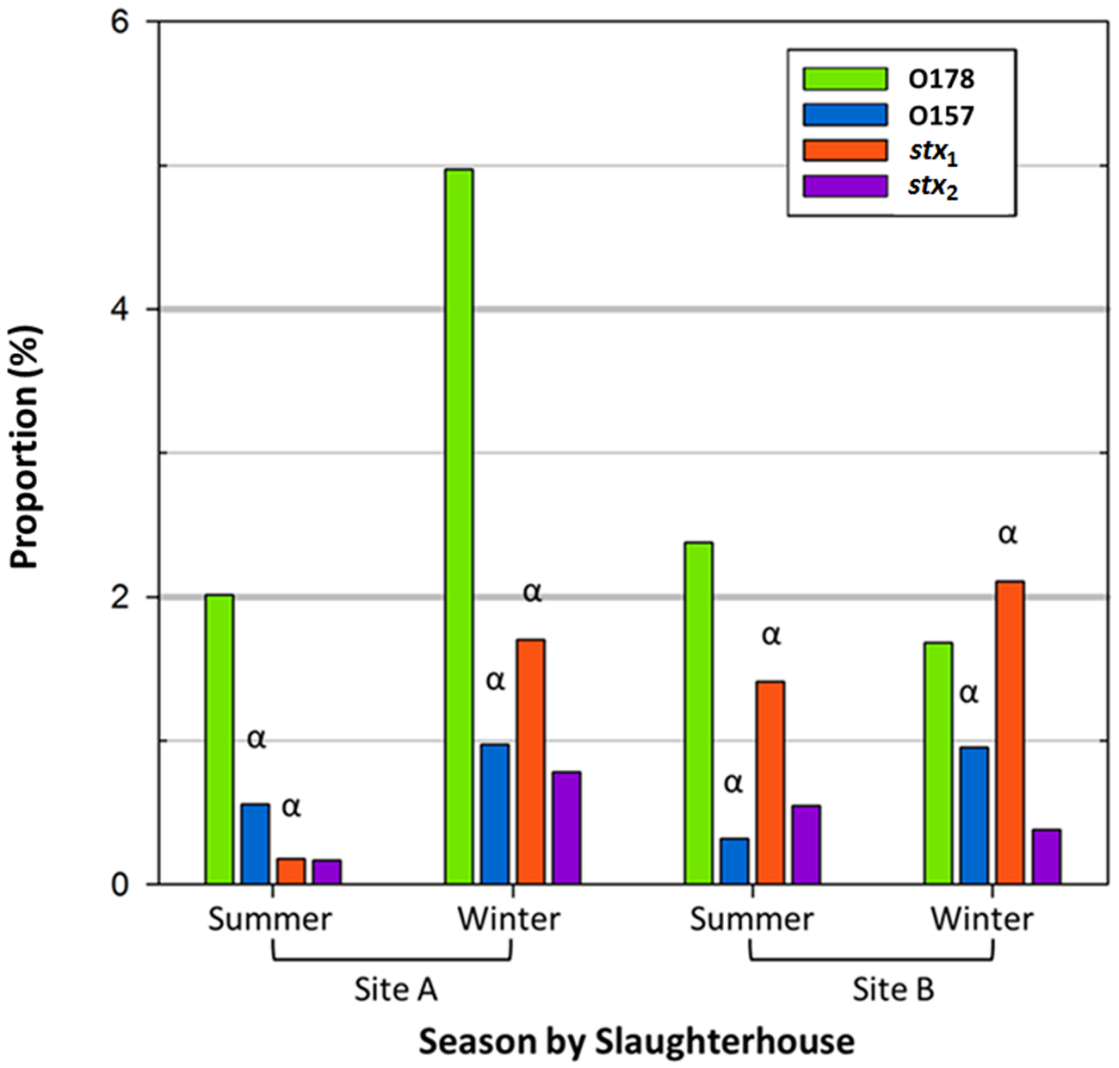
Comparison of the average proportion for O178, O157, shiga toxin (*stx*) 1 and 2 for the two sampling sites, A and B for each season based on the total generic *E*. *coli* count. Note: * Symbol: α above specific gene fragment denotes a significant difference in enumerations between summer and winter for that target (*P* < 0.05).

The individual O178, O157, *stx*_1_ and *stx*_2_ numerical data were used to calculate the proportion of gene fragments of O178, O157, *stx*_1_ and *stx*_2_ in the original DNA sample and compared to GEC (Fig 2). The proportion of O178 did not differ between slaughterhouses and ranged from ≤1% to 44% at the **A** site and ≤1% to 27% at the **B** site. In contrast, O157 proportion of GEC was higher at **A** (*P* < 0.05), ranging from ≤1% to 12% at **A** compared with ≤1% to 7% at **B**. The average proportion of O178 (2.8%) was greater (*P* < 0.05) than O157 (0.6%) (Fig 2) and varied by slaughterhouse and season (Fig 3). The *stx*_1_ proportion did not differ between slaughterhouses and ranged from ≤1% to 23% at **A** and ≤1% to 28% at **B** (Fig 2). Likewise, *stx*_2_ proportions were similar (*P* > 0.05) at **A** (≤1% to 13%) and at **B** (≤1% to 9%). In total, the average *stx*_1_ proportion in all samples was 1.4%, compared to 0.5% for *stx*_2_ (Fig 2).

The proportion of O178 did not differ significantly (*P* > 0.05) between seasons (Fig 3). In contrast, proportions of O157 versus GEC were higher (*P* < 0.05) in winter compared to summer at both sites (Fig 3). The proportions of *stx*_1_ were also higher (*P* < 0.05) in winter compared to summer for both slaughterhouses while the proportion of *stx*_2_ did not differ significantly (*P* > 0.05) across seasons (Fig 3).

#### Distribution of *stx*_1_ and *stx*_2_

**Fig 4:**
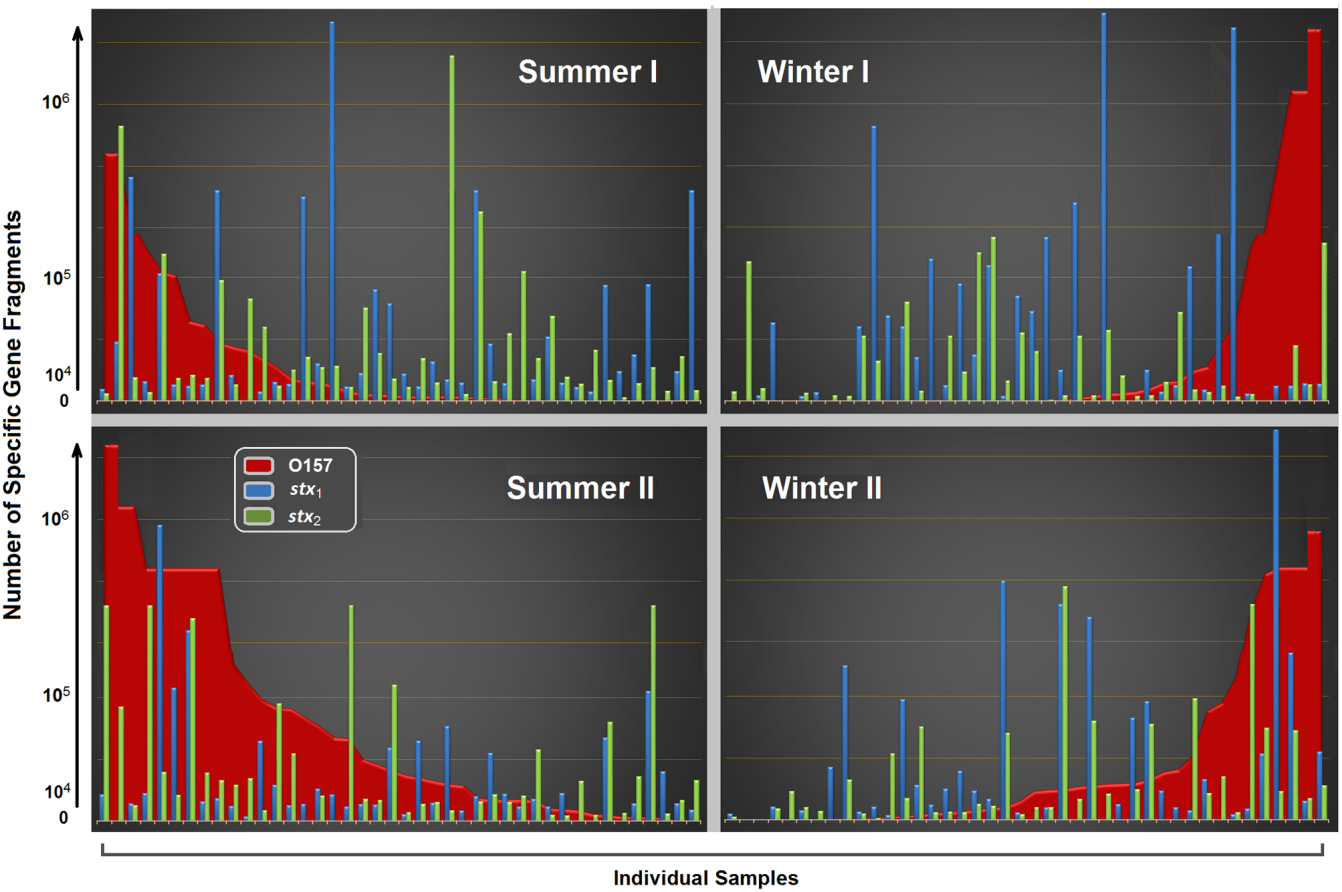
Total number of shiga toxin (*stx*) 1 and 2 specific gene fragments versus reduced O157 enumerations (-23% for non-STEC O157) for summer and winter across the sampling period. Each peak denotes a sample and the color (red = O157, blue = *stx*_1_ and green = *stx*_2_) indicates the target.

Examining the *stx*_1_ and *stx*_2_ enumerations versus the reduced (minus 23% non-STEC O157) O157 enumerations identified 67% samples where either one or both toxins had higher enumerations than could be accounted for by O157 (Fig 4).

## Discussion

### Conventional PCR screening of metagenomic DNA for O178

In the pool of samples analysed, *E*. *coli* O178 was identified in approximately half of samples (Table 2) as compared to O157 detection in 79% of samples. In contrast, only 7% of bovine rectum contents were positive for O178 in Argentina [20]. Masana et al. [21] revealed STEC O178:H19 as the most prevalent serotype (10.9%) among 35 different non-STEC serotypes from cattle feces and carcasses in beef slaughterhouses in Argentina. Pooling the feces from up to 45 cattle in our study might be one explanation for detecting higher prevalence compared to samples from individual animals. However, diagnostic limits may have underestimated detectable cell numbers in previous studies and/or the conditions in Alberta may favour increased O178 proliferation.

The presence of O178 in Canadian cattle was not unexpected as O178 has been isolated from cattle in other countries including Argentina [20], Spain [22] and Germany [5]. A study examining food sources in Germany, Switzerland and France identified O178:H19 in 80% of beef products, 10% dairy products and 10% goats and/or goat products, suggesting cattle are a major reservoir for this serotype [23].

In the present study, the occurrence of O178 showed significant differences between slaughterhouses **A** and **B**, and between the summer and winter seasons, suggesting that O178 prevalence is affected by animal origin (location) and seasonality. Consequently, we sought to determine whether the prevalence of O178 among the entire *E*. *coli* population was shifting across seasons and locations. Therefore, a representative subset of samples were further analysed to calculate the proportion of O178, O157, *stx*_1_ and *stx*_2_ versus total GEC populations by ddPCR and the distribution of *stx*_1_ and *stx*_2_ compared to O157.

### Droplet digital PCR

#### Total numbers of GEC, O178, O157, *stx*_1_ and *stx*_2_

We quantified and compared the total GEC in pooled cattle feces samples to the number of gene fragments from serogroups O178, O157 and *stx*_1_ and *stx*_2_ genes. Enumerations by slaughterhouse revealed that on average O178 and O157 were more numerous at **A** site, while GEC and *stx*_1_ and *stx*_2_ was greater at **B** site (Fig 1). Studies examining outbreaks [24] and infection [25] incidence of *E*. *coli* O157 in the United States identified a geographic trend with a greater occurrence in northern states compared to southern states. Contrary to Heiman et al. [24] and Sodha et al. [25] our data for feedlot origin at both slaughterhouses showed no discernable geographic differences between the animals at both locations but revealed divergences in feedlots shipping cattle to each slaughterhouse [17]. Ultimately, individual on-farm management factors may have a greater impact on the identified *E*. *coli* subtypes than does geographic location, although locational differences in prevalence of *E*. *coli* serogroups has been reported [26].

#### Proportion of O178, O157, *stx*_1_ and *stx*_2_

Further analysis of the bacterial enumerations was used to compare the proportion of *E*. *coli* O178, O157, *stx*_1_ and *stx*_2_ to total GEC. Overall, the average proportion of O178 was 4-times higher compared to O157 across all samples (Fig 2). These results are in accordance with Tanaro et al. [20] and Masana et al. [21] which found O178 to be either the most prevalent or among the most predominant serogroups in cattle in Argentina. Our data indicate that in Alberta, populations of O178 are also more numerous than those of O157. Miko et al. [5] reasoned that cattle feedlot management practices in Argentina may cause the emergence of O178 strains compared to grazing fed cattle. The greater proportion of O178 at both slaughterhouses compared to O157 might demonstrate a similar relationship with national standards in animal management selecting for specific microflora and/or suggesting feedlot animals are predisposed to greater O178 carriage compared to grazing animals. A review by Karmali [27], discussed that in North America infections related to STEC O157:H7 have decreased over the last two decades, mainly due to enhancements in food safety. However, Karmali [27] noted that despite these improvements, severe diseases associated with non-O157 STEC and emerging hybrid STEC strains are increasing. Ultimately, O178 may have always been relatively abundant, but were not noticed in earlier investigations.

More than 400 serotypes of STEC have been identified in cattle [22] and, both *E*. *coli* serogroups (O157 and O178) accounted for 3.4% of the total *E*. *coli* cells while the remaining 96.6% *E*. *coli* present were not tested for O-serogroup assignments (Fig 2). High throughput genome sequencing technologies [28] may substitute for current sub-typing gaps in the future.

The average proportion of *stx*_1_ was 3-times greater than *stx*_2_ across all samples (Fig 2) and reflects data on *stx*_1_ being more frequently isolated from patients in Alberta [29]. Conversely, other studies [30, 31, 32], found *stx*_2_ to be more prevalent in cattle samples, in Iran, Korea and the United States, respectively. Differences in proportion for *stx*_1_ versus *stx*_2_ may be geographically based or may be due to the sample size in this study as animals from different feedlots have dissimilar Shiga-toxin profiles [33].

Approximately 1.9% of the total *E*. *coli* population in our study carried either *stx*_1_ and/or *stx*_2_ (Fig 2). This leaves a reasonable cause for concern as an infectious dose for O157:H7 of 100 CFU [34] is considerably lower than the STEC numbers identified in our samples. As virulence genes may be transferred to a previously *stx*-negative population [35] 98.1% of the GEC in our study were potentially available as hosts. A “new” STEC was recently seen in Germany when an enteroaggregative O104:H4 acquired the *stx*_2_ gene resulting in a particularly virulent strain [36] and in France with the emergence of an O80:H2 hybrid [37].

Comparing the average proportion of *E*. *coli* O178 by season at each slaughterhouse showed no significant differences (Fig 3). A previous study [20] examined the prevalence of non-O157 in feces across different seasons and reported the highest prevalence of non-O157 STEC in winter (52.7%) compared to summer (36.1%). The proportion of O178 at slaughterhouse **A** followed a similar trend as the previous report [20] with greater proportions in winter compared to summer. The opposite trend was revealed at site **B**, although overall differences in seasonality were not identified. Food safety concerns for consumers are considered high during summer months due to increased O157 prevalence [38, 39] but our O178 numerical data revealed equal year-round numbers even though prevalence based on conventional PCR was higher for O178 in the winter than in the summer.

In comparison to O178, *E*. *coli* O157 proportion at both slaughterhouses was lower in summer versus winter (Fig 3). Our data contrasts with earlier studies [38, 39] which reported increased seasonal shedding of *E*. *coli* O157 during summer compared to winter which is thought to be correlated with increased O157 infections in summer [25]. Our finding of increased O157 proportions during winter months do not support the elevated numbers of infections in summer which might also be attributed to factors such as warm ambient temperatures or food handling practices [25], and/or an increased consumption of barbequed/grilled meat products [40]. However, the previous reports determined presence/absence and did not enumerate the total O157 *E*. *coli* population. The discrepancies between qualitative and quantitative data require further attention to evaluate the potential of food safety risks.

Comparing the average *stx*_1_ versus *stx*_2_ proportion among the two sampling sites by season demonstrated that seasonality was associated with *stx*_1_ (higher in winter, lower in summer) but not for *stx*_2_ (Fig 3). Both of our results are contradictory to Dewsbury et al. [41] findings that *stx*_1_ and *stx*_2_ were only present in the summer while we found *stx*_1_ and *stx*_2_ were present in both seasons with *stx*_1_ having significantly higher proportions in winter compared to summer. Dewsbury et al. [41] screened isolates found in feces after immunomagnetic separation, different from our study which examined the total DNA from all bacteria present in the collected feces. Comparing methodologies, Dewsbury et al. [41] supplemented media with antibiotics which may have altered the microflora detected in feces [42, 43].

#### Distribution of *stx*_1_ and *stx*_2_

Examining the distribution of *stx*_1_ and *stx*_2_ compared to O157 demonstrated several high-*stx*-events across seasons which cannot be attributed to O157 (Fig 4) and likely belong in part to the non-O157 STEC identified by Stanford et al. [17]. Cattle termed “super-shedders” defecate >10^4^ CFU/g of O157 [8] and are considered the main source of *E*. *coli* O157 contamination [44]. Data reviewed by Chase-Topping et al. [44] estimated that 8–9% of cattle are “super-shedders” but account for ≥96% of the bacteria shed. The sampling days where toxin numbers exceeded O157 numbers suggests that virulent but unidentified O-groups are being shed at high concentrations (Fig 4). Enumeration data also suggests that O178 may be super-shed by individual cattle based on O178 being more prevalent at site **B** but in higher numbers at **A** site (Table 2 & Fig 1). “Super-shedders” are thought to have a disproportionate impact on transmission along the farm-to-fork continuum and super-shedding is not limited to O157 as STEC O26 “super-shedders” have also been identified [8]. The discovery of peaks of *stx* amplicons not attributed to O157 suggests that it may be worthwhile to identify STEC “super-shedders” using virulence markers instead of serogroups as super-shedding events with uncommon STEC may be missed.

Droplet digital PCR technology was used in this study to quantify the total bacterial populations as well as individual strains by absolute quantification of genetic markers. An earlier study [45] found that ddPCR provided absolute quantification of STEC without the need for standards and that ddPCR is robust to inhibition which is often a concern screening environmental samples and/or using qualitative assays. Another study [46] used ddPCR to measure the qPCR standards used to screen pathogen loads to enhance their quantification and stated that ddPCR had superior accuracy than qPCR. Here, ddPCR successfully quantified the total *E*. *coli* population as well as specific *E*. *coli* O-serogroups, *stx*_1_ and *stx*_2_ in feces samples. The individual proportions generated by ddPCR identified various differences in location and seasonality and demonstrated a different trend in seasonality for O157 other than previously reported and contradicted the prevalence data for O178 at Alberta slaughterhouses. DdPCR data also identified peaks in *stx*_1_ and *stx*_2_ not associated with O157 that may indicate “super-shedders” of STEC other than O157. Data from ddPCR showed insight into the *E*. *coli* population pattern as part of the cattle microbiome composition. Future studies using ddPCR may further elucidate differences in trends between quantitative and qualitative data and help guide mitigation strategies for STEC by identifying periods of heightened virulence.

## Conclusions

Our data show that O178 is a subset of the total *E*. *coli* population present in Alberta cattle feces. Both, *stx*_1_ and/or *stx*_2_ were present in almost all cattle feces tested. Our data suggest that besides virulent O157, STEC “super-shedders”, not exclusively attributed to O157 exist. Overall, the data illustrate that food safety surveillance should focus on monitoring virulence factors instead of serogroup screening. Emerging pathogens are a global threat to the food industry, challenging current food safety protocols and taxing healthcare resources due to outbreaks and/or particularly virulent strains of *E*. *coli*. Pathogens are evolving by continuous host and environmental adaptation. Further understanding regarding their emergence using novel technologies may help elucidate approaches to reduce health risks along the farm-to-fork continuum.

## Supporting information

S1 Fig One-Dimensional snapshot of one ddPCR data set detecting O178 indicating the event number (droplets) versus the fluorescence amplitude shows the separation between positive droplets (blue) and the negative droplets (black). Droplets above the red threshold line (calculated by Quantasoft) are positive and those below negative.
(TIF)
