# Supplementary figures and images for: Quantitative surveillance of shiga toxins 1 and 2, Escherichia coli O178 and O157 in feces of western-Canadian slaughter cattle enumerated by droplet digital PCR with a focus on seasonality and slaughterhouse location
Source: PLoS One. 2018 Apr 12;13(4):e0195880. doi: 10.1371/journal.pone.0195880 (PMC5897018; doi:10.1371/journal.pone.0195880)

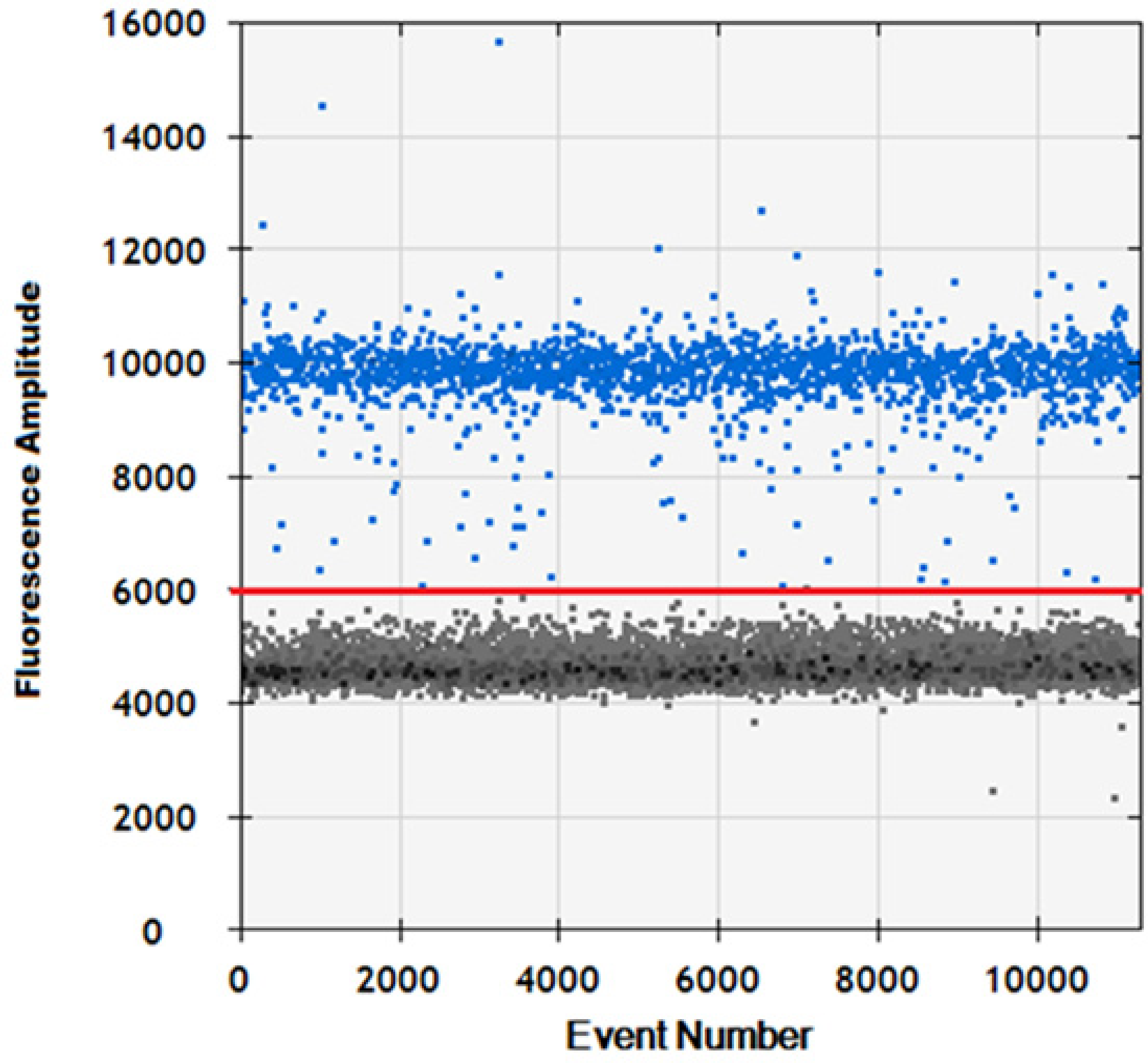

Supplement: S1 Fig — Droplets above the red threshold line (calculated by Quantasoft) are positive and those below negative. (TIF) [file pone.0195880.s001.tif]
